# Two closely spaced mutations *in cis* result in Ullrich congenital muscular dystrophy

**DOI:** 10.1038/s41439-019-0052-z

**Published:** 2019-04-26

**Authors:** Hideki Shimomura, Tomoko Lee, Yasuhiko Tanaka, Hiroyuki Awano, Kyoko Itoh, Ichizo Nishino, Yasuhiro Takeshima

**Affiliations:** 1 Department of Pediatrics, Hyogo College of Medicine, Nishinomiya, Japan; 2 Department of Pediatrics, Kobe University Graduate School of Medicine, Kobe, Japan; 3 Department of Pathology and Applied Neurobiology, Graduate School of Medical Science, Kyoto Prefectural University of Medicine, Kyoto, Japan; 4 Department of Neuromuscular Research, National Institute of Neuroscience, National Center of Neurology and Psychiatry, Tokyo, Japan

**Keywords:** Neuromuscular disease, Disease genetics

## Abstract

A 2-year-old boy was diagnosed with Ullrich congenital muscular dystrophy (UCMD) by muscle biopsy. COL6A3 gene analysis by next-generation sequencing revealed two heterozygous splice-site mutations (c.6283-1 G > G/T and c.6310-2 A > A/T), whereas normal mRNA was produced. Genomic DNA analysis revealed two mutations located on the same allele; however, no mutation was detected in either parent. These results indicated that two closely spaced de novo mutations resulted in the autosomal dominant UCMD.

Ullrich congenital muscular dystrophy (UCMD), a collagen type VI-related disorder resulting from protein defects in the extracellular matrix, is characterized by muscle weakness, hypotonia, proximal joint contractures, and marked hyperlaxity of the distal joints (MIM No. 254090). Another example of a collagen type VI-related disorder is Bethlem myopathy (BM; MIM No. 158810). These conditions arise from mutations in *COL6A1* and *COL6A2* situated head-to-tail on 21q22.3, as well as in *COL6A3* located on 2q37.3. UCMD and BM were previously considered to be an autosomal recessive condition and an autosomal dominant condition, respectively. However, cases of autosomal dominant UCMD^1^ and autosomal recessive BM^2^ have also been reported. Currently, both UCMD and BM are known to occur either via autosomal recessive or autosomal dominant mechanisms. Although homozygous and compound heterozygous mutations have been described in patients with both UCMD and BM, no cases with closely spaced de novo mutations *in cis* have been reported. Here, we report a patient with UCMD carrying two closely spaced de novo mutations in the same *COL6A3* allele.

A 2-year-old boy was born normally after 35 weeks of gestation. He exhibited mild developmental delay, with head control at 6 months and walking at 15 months of age. His serum creatine kinase level was 189 U/L. He presented marked distal joint looseness, diminished deep tendon reflexes, and a waddling gait. At 6 years of age, he could not walk by himself. Histochemical analysis and collagen VI immunostaining verified UCMD diagnosis. Next-generation sequencing of the genomic DNA revealed two heterozygous splice-site mutations in *COL6A3* (viz., c.6283-1 G > G/T and c.6310-2 A > A/T), and mRNA analysis revealed normal as well as aberrantly spliced transcripts. These findings were previously reported in patient 7 in a study, which suggested a dominantly inherited condition^3^.

For *COL6A3* mRNA analysis, total RNA was isolated from thin-sliced muscle sections obtained from frozen biopsied muscle tissue. Reverse-transcription polymerase chain reaction (RT-PCR) was employed using a forward primer that corresponded to a segment of exon 15 (c15F: 5′-GACAACATTGCCGAGAAAGC-3′) and a reverse primer that corresponded to a segment of exon 21 (c21R: 5′-TCGAATCCCAACATCTCCTC-3′). The PCR products were separated by electrophoresis on a 3% agarose gel. To detect and semiquantify the minor RT-PCR products, the amplified products were analyzed by capillary electrophoresis (3500xL Genetic Analyzer; Applied Biosystems) after a 25-cycle PCR amplification using an unlabeled c15F primer and FAM-labeled c21R primer. The amount of each product was quantified by measuring the peak areas. The RT-PCR products were then subjected to sequencing after subcloning into a pT7Blue T-vector as described previously^4^.

To determine whether the two mutations were located on the same allele, the nucleotide sequence of the PCR products amplified from genomic DNA using a forward primer on intron 17 (g17F: 5′-AACATTCAAATGGGGTGGAG-3′) and reverse primer on intron 19 (g19R: 5′-GAACCAAAAGCAGTTTGGACTT-3′) was determined after subcloning into the pT7Blue T-vector.

To characterize the *COL6A3* nucleotide change in the patient’s parents, a region encompassing exons 18 and 19 was PCR-amplified from genomic DNA using the g17F and g19R primers. The PCR products were directly sequenced with the BigDye Terminator Cycle Sequencing kit (Amersham Biosciences, Piscataway, NJ, USA) using the automatic DNA sequencer 3500xL Genetic Analyzer.

To analyze the impact of the mutations on splicing probability, we used the in silico prediction tool MaxEntScan with the Weight Matrix Model (http://genes.mit.edu/burgelab/maxent/Xmaxentscan_scoreseq_acc.html). A high score indicated a high possibility of the sequence being a splicing site^5^. The Shapiro splicing probability matrix scores (Shapiro’s score) were calculated as described previously^6^. Additionally, in silico analysis for the number of exonic splicing enhancers (ESEs) and intronic splicing enhancers (ISEs) was also performed using the ACESCAN2 Web Server (http://genes.mit.edu/acescan2/)^7^.

Based on previous results, we evaluated *COL6A3* mRNA semiquantitatively, including the minor products, and muscle *COL6A3* mRNA was analyzed using capillary electrophoresis. Analysis of the muscle-expressing *COL6A3* mRNA revealed three kinds of products: a normal mRNA and two larger mRNAs (Fig. 1a, b). Nucleotide sequencing after subcloning revealed that exon 18 was skipped and that either a 37-nt or a 42-nt sequence of the latter part of intron 18 was inserted into the two larger mRNAs (Fig. 1c). Semiquantitative analysis by capillary electrophoresis revealed that 0.4% and 48.6% of mRNAs were included in the 37- and 42-nt sequences, respectively (Fig. 1b).

**Fig. 1:**
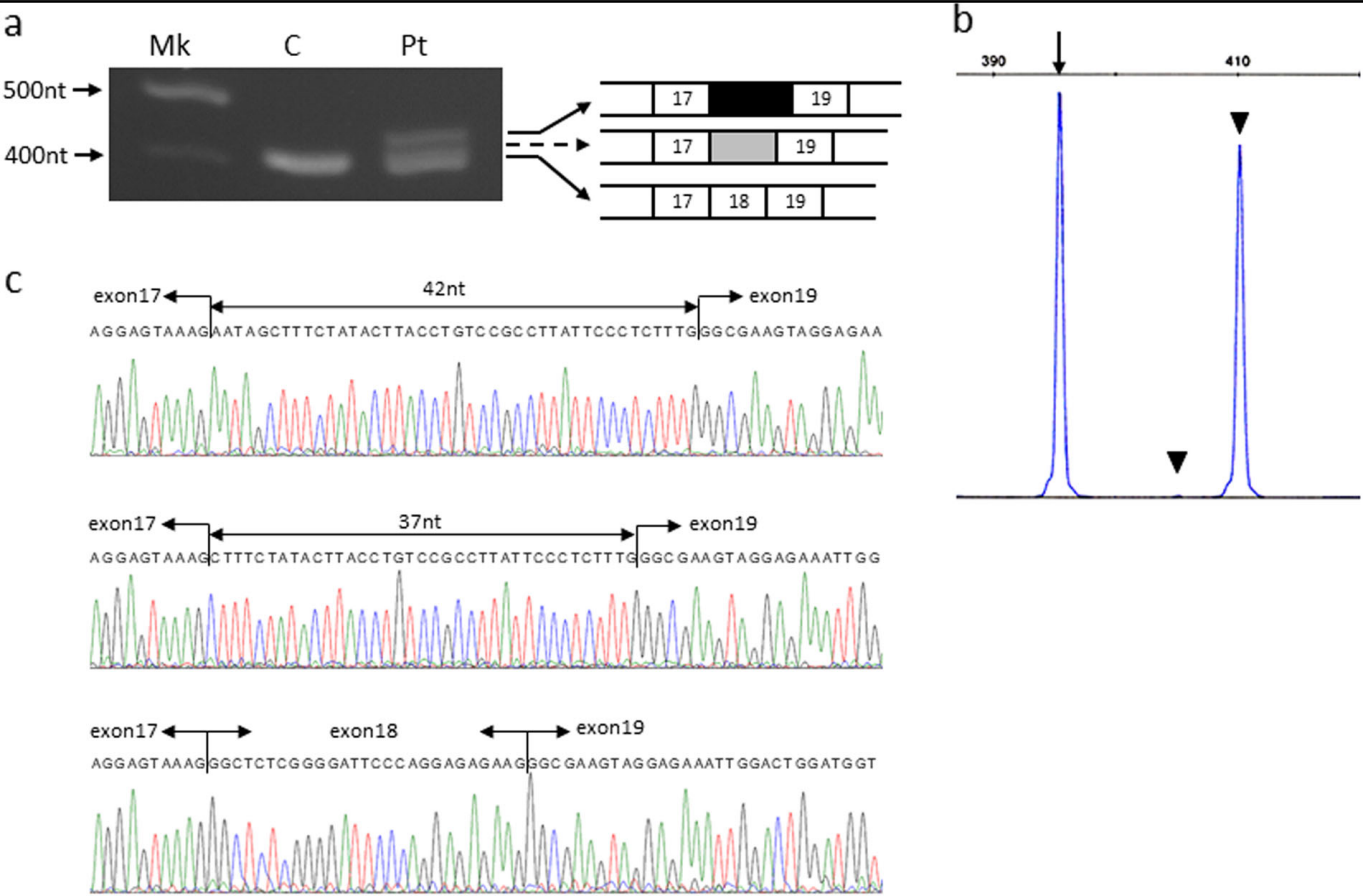
Analysis of *COL6A3* mRNA in the index case. **a** Agarose gel electrophoresis of the amplified products encompassing exons 15–21. Fragments encompassing exons 15–21 were amplified using cDNA prepared from the muscle samples of a control individual (**c**) and the patient (Pt). Mk indicates the DNA marker. An additional larger band was visualized in the patient muscle cDNA. The structure of each product is shown schematically on the right side. The numbers in the boxes indicate the exon number, and black and gray boxes represent the 42- and 37-nt sequences of the latter part of intron 18, respectively. An additional band from the 37-nt insertion product was not detected (dotted arrow). **b** Capillary gel electrophoresis of the amplified products encompassing exons 15–21 from the muscle cDNA of the patient. The trace displayed three kinds of cDNA. The shortest product was the same size as that of normal cDNA (arrow), whereas 0.4% and 48.6% of the products were larger than the normal size (arrowhead). **c** Sequences of the three cDNA fragments from the patient’s muscle tissue. The shortest product revealed a normal nucleotide sequence (bottom), whereas in the larger-sized products, exon 18 was skipped, and the 37- and 42-nt sequences of the latter part of intron 18 were inserted (middle and top, respectively)

The genomic DNA sequence was analyzed after subcloning. The results revealed that one allele had a normal sequence, whereas the two closely spaced mutations on the other allele were separated by 137 nt. These mutations were not identified in the boy’s parents. The findings concluded that there were two closely spaced de novo mutations in the same allele of *COL6A3*, where c.6283-1 G > T resulted in the skipping of exon 18, and c.6310-2 A > T activated the cryptic splice sites in the 37- and 42-nt sequences upstream of exon 19 (Fig. 2).

**Fig. 2:**
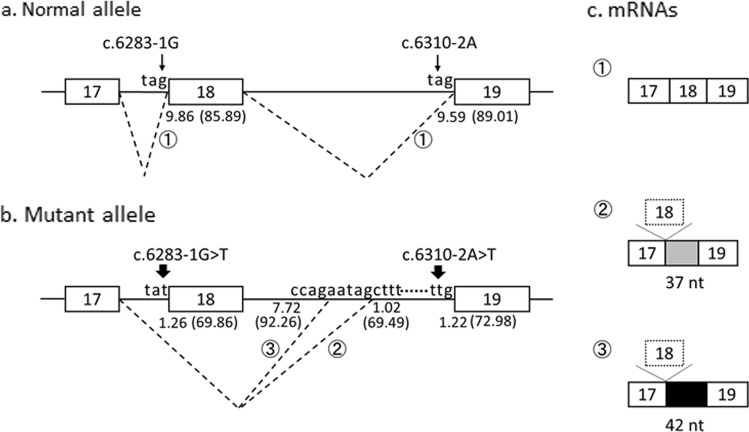
Schematic representation of the splicing patterns identified in the index case. Thin and bold arrows represent mutations. **a** Normal *COL6A3* allele in the index case. **b** Mutant *COL6A3* allele with two closely spaced mutations. **c** *COL6A3* mRNAs. The dotted lines indicate the splicing events. Splicing pattern ①: Normal mRNA was produced from the normal allele (**a**, **c**①). Splicing patterns ② and ③: The c.6283-1 G > T mutation resulted in the skipping of exon 18, whereas the c.6310-2 A > T mutation activated the cryptic splice sites in the 37- and 42-nt sequences upstream of exon 19. The MaxEntScan and Shapiro’s score (in parentheses) of each splice acceptor site are depicted below each authentic and cryptic splice acceptor site. Both scores of the authentic splice acceptor sites were decreased by these mutations. Boxes and lines indicate exons and introns, respectively, and the numbers in the boxes indicate the exon number. Gray and black boxes represent the 37- and 42-nt sequences of the latter part of intron 18, respectively, and the dotted box indicates the skipped exon. Partial nucleotide sequences of the introns are shown above the line

The MaxEntScan score of the splice acceptor site of exon 18 was 9.86 and that of exon 19 was 9.59. As a result of mutation, these scores changed to 1.26 in exon 18 and 1.22 in exon 19. The scores of the cryptic splice sites 37 and 42 upstream of exon 19 were 7.72 and 1.02, respectively (Fig. 2). The Shapiro’s score of these splice sites was evaluated as shown in Fig. 2. In silico analysis of ESEs and ISEs revealed that the number of ESEs in exon 18 was 4 and that in exon 19 was 10, while the number of ISEs in intron 18 was 12 and that in intron 19 was 6.

In this report, a case of autosomal dominant UCMD with two closely spaced de novo mutations in the same allele was identified for the first time. Closely spaced multiple mutations (CSMMs) were classified into two groups: those separated by <100 nt and those separated by >100 nt^8^. Lampe et al. (2005, 2008) reported a large number of mutations related to collagen type VI-related disorders, all of which were single nucleotide substitutions, but none were CSMMs. Chen et al. reviewed 58 cases of various disorders with CSMMs separated by <100 nt^8^. In the context of multiple mutations, a few cases of UCMD with compound heterozygous mutations were reported^9,10^.

In our case, both mutations were located at splice acceptor sites. The first mutation, c.6283-1 G > T, was located at the splice acceptor site of intron 17 and was predicted to cause the skipping of exon 18. The second mutation, c.6310-2 A > T, was located at the splice acceptor site of intron 18. A mutation located at the splice site towards the N-terminal end of the triple helical domain was previously reported to cause single exon skipping^10^. Therefore, c.6310-2 A > T was also predicted to cause the skipping of exon 19. Unexpectedly, c.6310-2 A > T caused the activation of the cryptic splice sites. In this case, the resultant mRNAs from c.6283-1 G > T and c.6310-2 A > T skipped exon 18 and preserved exon 19 while inserting the latter part of the intron 18 sequence. We assumed that this might be due to the difference in the number of ESEs and ISEs. In silico analysis revealed that ESE numbers in exon 18 were fewer than those in exon 19. In contrast, the ISE numbers in intron 18 were greater than those in intron 19. Thus, this analysis could not fully explain the skipping of exon 18 and the preservation of exon 19. Further research is needed to explain this phenomenon.

The results of electrophoresis and semiquantitative analysis by capillary electrophoresis revealed that the major mRNA products included the 42-nt sequence that maintained the reading frame. Since the amount of minor product was markedly small, we could not detect any bands. Thus, the findings suggest that the abnormal protein from the major product acts in a dominant fashion and that the minor product is too scarce to function.

Traditionally, UCMD was considered to be an autosomal recessive condition with severe clinical manifestations, whereas BM was considered to be an autosomal dominant condition with mild clinical outcomes. However, whether a condition is autosomal dominant or autosomal recessive is distinguished from the mutation patterns. Usually, cases that demonstrate heterozygous mutations are considered to be dominant conditions, whereas homozygous and compound heterozygous mutations are considered to be recessive conditions. However, in cases with two mutations, it is sometimes unclear from published reports whether these mutations are located in the same allele or in different alleles. Therefore, we suggest the existence of more undiagnosed dominant cases with multiple mutations.

## Data Availability

The relevant data from this Data Report are hosted at the Human Genome Variation Database at 10.6084/m9.figshare.hgv.2576
